# miR-198 Represses the Proliferation of HaCaT Cells by Targeting Cyclin D2

**DOI:** 10.3390/ijms160817018

**Published:** 2015-07-27

**Authors:** Jian Wang, Guorong Dan, Tao Shangguan, Han Hao, Ran Tang, Kaige Peng, Jiqing Zhao, Huiqin Sun, Zhongmin Zou

**Affiliations:** 1 Institute of Toxicology, School of Preventive Medicine, the Third Military Medical University, 30 Gaotanyan Street, Shapingba District, Chongqing 400038, China; E-Mails: wellginger1987@hotmail.com (J.W.); liroy@163.com (G.D.); shangtaott@126.com (T.S.); pkgtmmu@163.com (K.P.); zhaojiqing392@sohu.com (J.Z.); huiqinsun02@163.com (H.S.); 2 The 17th Student Battalion, School of Preventive Medicine, the Third Military Medical University, 30 Gaotanyan Street, Shapingba District, Chongqing 400038, China; E-Mails: haohan2016@126.com (H.H.); tangran2016@126.com (R.T.)

**Keywords:** miR-198, cyclin D2, HaCaT, cell proliferation

## Abstract

Background: MiR-198 has been considered as an inhibitor of cell proliferation, invasion, migration and a promoter of apoptosis in most cancer cells, while its effect on non-cancer cells is poorly understood. Methods: The effect of miR-198 transfection on HaCaT cell proliferation was firstly detected using Cell Count Kit-8 and the cell cycle progression was analyzed by flow cytometry. Using bioinformatics analyses and luciferase assay, a new target of miR-198 was searched and identified. Then, the effect of the new target gene of miR-198 on cell proliferation and cell cycle was also detected. Results: Here we showed that miR-198 directly bound to the 3′-UTR of CCND2 mRNA, which was a key regulator in cell cycle progression. Overexpressed miR-198 repressed CCND2 expression at mRNA and protein levels and subsequently led to cell proliferation inhibition and cell cycle arrest in the G1 phase. Transfection ofSiCCND2 in HaCaT cells showed similar inhibitory effects on cell proliferation and cell cycle progression. Conclusion: In conclusion, we have identified that miR-198 inhibited HaCaT cell proliferation by directly targeting CCND2.

## 1. Introduction

miRNAs are small non-coding RNAs ranging in length from 19 to 25 nucleotides, which regulate gene expression usually by targeting the 3′-UTR of mRNA for translation repression, degradation or both [1]. It is estimated that about 60% genes can be regulated by miRNAs [2]. MiR-198 has been recently reported as a tumor suppressor by repressing mitogenic and motogenic pathways and diminishing cell growth in hepatocellular carcinoma [3,4], multiple myeloma [5], pancreatic adenocarcinoma [6], colorectal cancer [7,8] and lung cancer [9]. Fucosyltransferase 8 (FUT8) [7], E74-like factor 3 (ElF3) [8] and fibroblast growth factor receptor 1 (FGFR1) [9] are all confirmed to be the targets of miR-198. Though the elevated expression ofmiR-198 significantly represses re-epithelialization and wound healing in non-healing chronic diabetic ulcers [10], the potential effect of miR-198 on cell proliferation is poorly understood in non-cancer tissue or cells. D-type cyclins are known to play critical roles in cell cycle progression [11]. Three D-type cyclins, cyclin D1, D2 and D3, are encoded by distinct genes, but show significant amino-acid similarity. Among them, cyclin D2 (CCND2) is a key player in cell cycle progression from the G1 phase to S phase [12,13]. In 2008, Liu *et al.* [14] found miR-133a regulated cardiomyocyte proliferation by targeting CCND2, and this was the first research concerning the relationship between miRNA and CCND2. Then, various studies on different cells have shown miR-26a [15], miR-302b, miR-497 [16], miR-133b [17], miR-1, miR-206, miR-29 [18], and miR-603 [19] could regulate cell proliferation by targeting CCND2.

Here, we show that miR-198 represses the proliferation of HaCaT cells, a keratinocyte cell line, by targeting cyclin D2. MiR-198 may be a key regulator in keratinocyte cell growth.

## 2. Results and Discussion

### 2.1. miR-198 Represses the Proliferation of Cells

**Figure 1:**
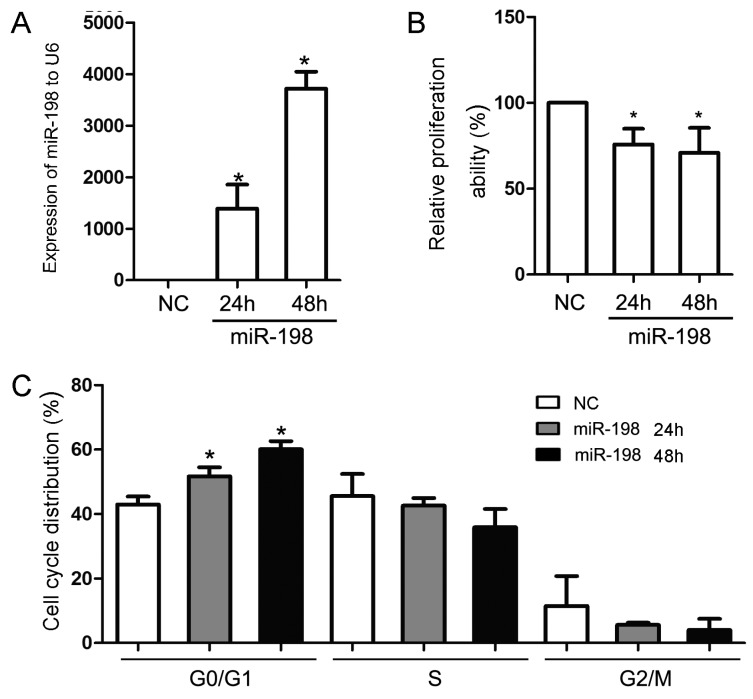
MiR-198 transfection inhibited HaCaT cell proliferation by cell cycle arrest in G1 phase. (**A**) MiR-198 mimic transfection led to a significantly elevated miR-198 expression in HaCaT cells both at 24 h (1390.00 ± 468.20 folds) and 48 h (3718.00 ± 329.40 folds); (**B**) Cell viability analysis showed that miR-198 mimic transfection inhibited HaCaT cell proliferation at 24 h (75.77% ± 9.14%) and 48 h (70.94% ± 14.54%); (**C**) Flow CytoMeter analysis showed the effect of miR-198 transfection on cell cycle progression, and G1 phase arrest was obvious at 24 h (51.71% ± 2.81%) and 48 h (60.07% ± 2.54%) after the transfection when compared with the negative control (42.98% ± 2.48%). (NC, negative control. * Compared with NC, *p* < 0.05).

The effect of overexpressed miR-198 on HaCaT cell proliferation was firstly evaluated. After transfecting with an miR-198 mimic, the expression of miR-198 in HaCaT cells elevated significantly at both 24 h (1390.00 ± 468.20 folds) and 48 h (3718.00 ± 329.40 folds) compared with that transfected with the mimic negative control (Figure 1A). Cell viability analysis showed that the proliferation of HaCaT cells were dramatically inhibited to 75.77% ± 9.14% and 70.94% ± 14.54% at 24 and 48 h, respectively, compared with the controls (Figure 1B). Further detection by Flow CytoMeter (FCM) revealed that elevated expression of miR-198 lead to G1 phase arrest as early as 24 h (51.71% ± 2.81%) after the transfection (control group, 42.98% ± 2.48%), and even more increased in G1 phase distribution at 48 h (60.07% ± 2.54%) (Figure 1C).

### 2.2. Prediction of miR-198 Binding Sites in the 3′-UTR of CCND2 mRNA

**Figure 2:**
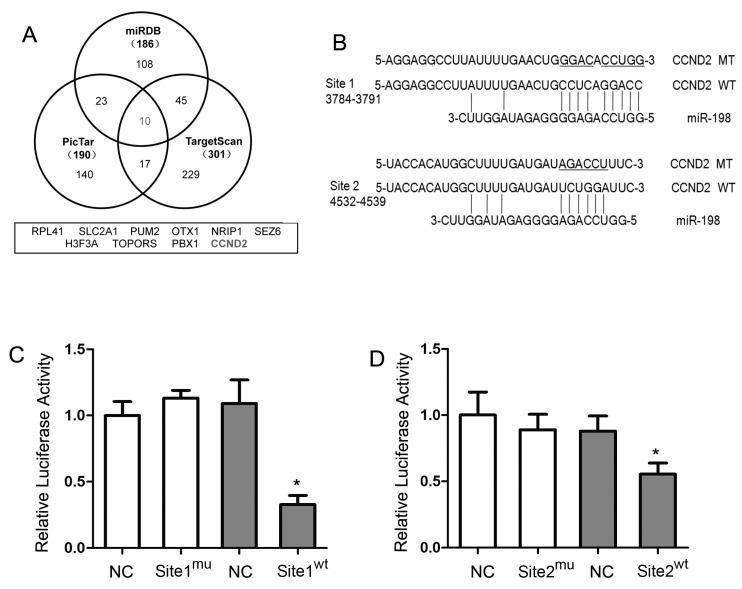
MiR-198 directly bound to the 3′-UTR of CCND2 mRNA. (**A**) Bioinformatics analyses showed that 10 potential target genes of miR-198 were predicted by three different databases; (**B**) The two distinct predicted binding sites of miR-198 in the 3′-UTR of CCND2 mRNA were allocated, and the fragments containing either mutated binding site were amplified according to the mature miR-198 sequence; (**C**) In pMIR-REPORT™ vector, CCND2 mRNA 3′-UTRfragment containing either the wild type or the mutated Site 1 was fused downstream the reporter gene. When the vectors were cotransfected with miR-198 mimic or mimic control, and the relative luciferase activity, normalised by β-gal, was significantly suppressed in vector with wild type Site 1 than that with mutated Site 1 (32.80% ± 6.89%); (**D**) In the presence of wild type Site 2, not the mutated one, miR-198 was able to significantly inhibit luciferase activity although to a less extent than wild type Site 1 (55.39% ± 8.48%). (NC, negative control; WT, wide-type; MT, mutated-type. * Compared with NC, *p* < 0.05).

In order to identify the downstream mRNA targets of miR-198, three independent online databases, Pic Tar, Target Scan and the miR DataBase (miRDB) were used to predict the potential targets. Ten putative mRNAs were unanimously predicted by the three algorithms (Figure 2A), and among which, CCND2 was of particular interest since previous studies have reported its important role in the cell cycle progress. After analyzing the sequence of the 3′-UTR of CCND2 (5341 bp), two predicted binding sites of miR-198 were found at 3784–3791 (Site 1) and 4532–4539 (Site 2) respectively (Figure 2B).

### 2.3. Luciferase Assay of miR-198 and CCND2 3′-UTR in HaCaT Cells

To determine whether CCND2 is a target of miR-198, a luciferase reporter gene was fused to either the wide-type or the mutated CCND2 mRNA 3′-UTR, and was cotransfected with miR-198 mimic. Forced expression of miR-198 in HaCaT cells for 24 h reduced the activity of a luciferase reporter gene fused to wild-type CCND2 mRNA 3′-UTR, while the activity with mutated CCND2 mRNA 3′-UTR was not affected. Moreover, the binding Site 1 (Figure 2C) exhibited a more significant inhibition on luciferase activity than Site 2 (32.80% ± 6.89% *vs.* 55.39% ± 8.48%, Figure 2D).

### 2.4. Forced Expression of MiR-198 Reduces CCND2 Expression

**Figure 3:**
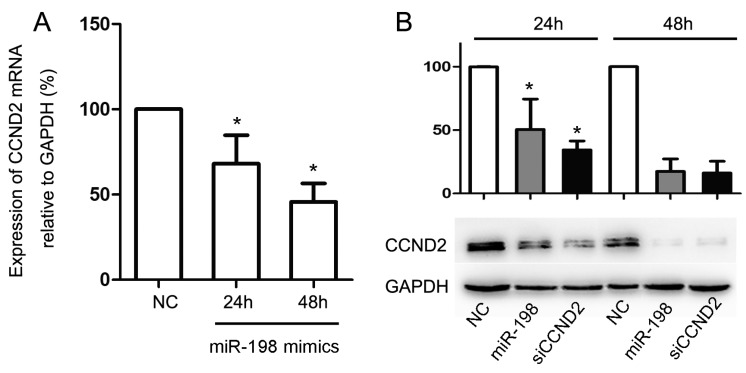
MiR-198 transfection repressed the mRNA and protein expression of CCND2. (**A**) MiR-198 mimic transfection reduced the expression of CCND2 mRNA at 24 h (68.09% ± 16.73%) and 48 h (45.68% ± 10.94%); (**B**) MiR-198 mimic or CCND2 siRNA transfection reduced the expression of CCND2 protein at 24 h (50.55% ± 24.04% or 34.45% ± 6.98%) and 48 h (17.38% ± 9.96% or 16.24% ± 9.23%). (NC, negative control. * Compared with NC, *p* < 0.05).

To verify if miR-198 expression affects the expression of CCND2 at both mRNA and protein levels, the miR-198 mimic its negative control were transfected to HaCaT cells. These cells were then harvested, and the expression of CCND2 at 24 and 48 h after the transfection was measured for both mRNA and protein by qPCR and Western Blot. As expected, the forced expression of miR-198 reduced the expression of CCND2 mRNA at 24 h (68.09% ± 16.73%), and the reduction was even more significant at 48 h (45.68% ± 10.94%, Figure 3A). A similar result was obtained when detecting the protein level of CCND2 (50.55% ± 24.04% at 24 h and 17.38% ± 9.96% at 48 h, Figure 3B).

### 2.5. CCND2 siRNA Transfection Represses the Proliferation of Cells

**Figure 4:**
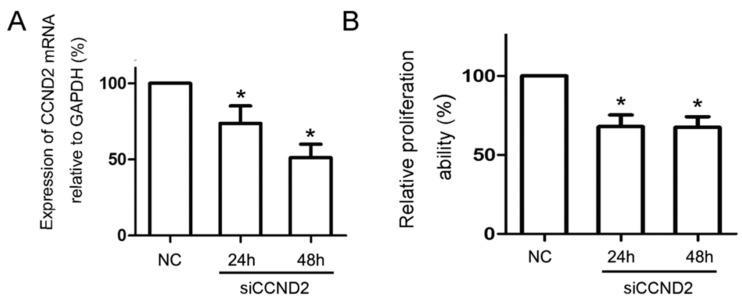

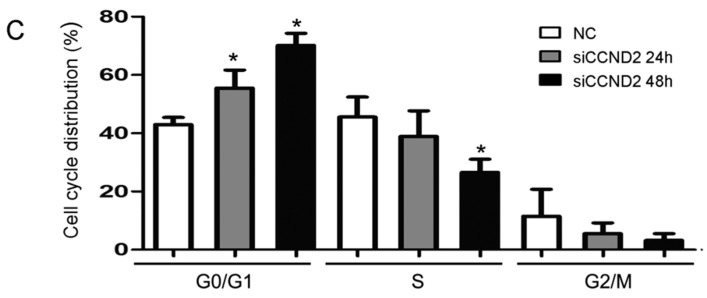
CCND2 siRNA transfection inhibited HaCaT cell proliferation by blocking cell cycle at G1 phase. (**A**) CCND2 siRNA transfection led to a significant reduction of mRNA expression of CCND2 in HaCaT cells both at 24 h (73.79% ± 11.45%) and 48 h (51.18% ± 8.85%); (**B**) Cell viability analysis showed that CCND2 siRNA transfection inhibited HaCaT cell proliferation at 24 h (67.98% ± 7.31%) and 48 h (67.45% ± 6.70%); (**C**) FCM analysis showed the effect of CCND2 siRNA transfection on cell cycle progression, and G1 phase arrest was obvious at 24 h (55.51% ± 6.18%) and 48 h (70.19% ± 4.10%) compared with negative control (42.98% ± 2.48%). (NC, negative control. * Compared with NC, *p* < 0.05).

Finally, the importance of CCND2 as a functional target of miR-198 was determined. HaCaT cells were transfected with CCND2 siRNA or scramble control for 24 and 48 h, and the mRNA (73.79% ± 11.45% at 24 h and 51.18% ± 8.85% at 48 h, Figure 4A) and protein expression (34.45% ± 6.98% at 24 h and 16.24% ± 9.23% at 48 h, Figure 3B) of CCND2 were both reduced significantly by CCND2 SiRNA transfection. CCK-8 assay and FCM were used to evaluate the proliferation ability and cell cycle distribution of the transfected HaCaT cells. The proliferation ability of HaCaT cells reduced to 67.98% ± 7.31% of the control at 24 h, and 67.45% ± 6.70% at 48 h (Figure 4B). FCM analysis showed that reduced expression of CCND2 lead to G1 phase arrest, and the percentages of cells in G1 phase increased from 42.98% ± 2.48% to 55.51% ± 6.18% at 24 h and to 70.19% ± 4.10% at 48 h (Figure 4C).

### 2.6. Discussion

miRNAs are firstly processed from coding or non-coding transcripts in the nucleus as primary miRNAs, and then they are transported to cytoplasm after being further processed into pre-miRNAs by cellular nucleases. In cytoplasm, pre-miRNAs are cut by Dicer into mature forms. These mature miRNAs combine to the RNA-induced silencing complex for further mRNA regulation. By directly binding to the 3′-UTR of mRNA, mature miRNAs promote the degradation and/or hinder the translation of targeted mRNA [20,21], and, finally, the protein expression are specifically repressed. miRNAs play key roles in both various biological processes and human disease development, and increasing research has indicated that miRNAs are involved in cell apoptosis, proliferation, differentiation, migration, and metastasis.

Since 2008, the decreased expression of miR-198 has been identified in most of the cancer types, such as papillary thyroid carcinoma [22], osteosarcoma [23], hepatocellular carcinoma [24], and pancreatic cancer [25]. Most of this research depicted the expression status of miR-198, but the effect of decreased or elevated expression of miR-198 was poorly understood. In 2011, Tan *et al.* [24] found that miR-198 was a novel suppressor of hepatocellular carcinoma cell (HCC) invasion by negative regulation of the HGF/c-MET pathway. In 2013, Chonglei *et al.* [5] found miR-198 was underexpressedin multiple myeloma (MM), and ectopic restoration of miR-198 significantly repressed MM cell proliferation, colony formation and migration. In hepatoma, central signal transducers of proliferation pathways were downregulated by miR-198. In contrast, genes mediating cellular adherence were highly upregulated by miR-198 [4]. Moreover, by targeting surviving [26] and fibroblast growth factor receptor 1 (FGFR1) [9], miR-198 promote cell apoptosis in human prostate cancer cell lines and lung cancer cells. Therefore, miR-198 may inhibit the growth of cancer cells by repressing cell proliferation, invasion, migration and promoting cell apoptosis in several cancer types. While in squamous cell carcinoma of tongue [27], retinoblastoma [28], prostate cancer [29], the expression of miR-198 was elevated, and this need to be further investigated.

Wound healing is a complex process involving the proliferation, migration and differentiation of unwounded keratinocyes nearby. In diabetes ulcers, a classic refractory wound healing, miR-198 expression was significantly upregulated and showed anti-migratory effect by targeting DIAPH1, PLAU and LAMC2 [10]. Considering most of the studies showed the effect of miR-198 on cancer cells, the investigation of its effect on non-cancer cells was needed. In the present study, miR-198 overexpression inhibited cell proliferation dramatically in keratinocyte cell line, HaCaT cells, and significant G1 phase arrest was detected by cell cycle analysis. In order to explain the inhibitory effect of miR-198, a screening for possible target of miR-198 was performed by bioinformatics analyses. Fortunately, CCND2 was identified as one of the potential targets with two predictive binding sites. Next, it was confirmed that the expression of CCND2 mRNA and protein all could be repressed by miR-198 overexpression. Using luciferase assay, one of the two potential binding sites of miR-198 on the 3′-UTR of CCND2 mRNA was identified as the main binding site. Further experiments should be done to test if an additive or synergistic effect exists between these two binding sites. Based on the identification of CCND2 as a new target of miR-198, we evaluated the function of CCND2m in cell proliferation and cell cycle control. The transfection of CCND2 SiRNA also led to a similar proliferation inhibition and G1 phase arrest in HaCaT cells as miR-198 overexpression. Our result showed that miR-198 inhibited keratinocyte proliferation by repressing one of its target genes CCND2. Future research may as well determine whether miR-198 will regulate different genes for cell invasion or migration inhibition.

## 3. Experimental Section

### 3.1. Cell Culture

HaCaT cells, a human skin epithelial cell line, were purchased from the cell bank of the Institute of Cell Biology, Chinese Academy of Science, Shanghai, and cultured in RPMI 1640 (HyClone, Logan, Utah, USA) plus 10% heat-inactivated fetal bovine serum (HyClone), 100 U/mL penicillin and 100 U/mL streptomycin (Beyotime, Beijing, China). All cells were maintained in a humidified incubator at 37 °C and 5% CO_2_.

### 3.2. RNA Oligonucleotides and Transfection

The miRNA mimic and siRNAs were synthesized by Ribo Bio (Guangzhou, China). MiR-198 mimic were synthetic duplexes representing mature miRNAs, and the mimic negative control sequence was supplied by Ribo Bio (Guangzhou, China). The oligonucleotide sequence of CCND2 siRNA was as follows: 5′-UGCUCCUCAAUAGCCUGCAGCAGUA-3′. SiRNA and miRNA transfections were performed using Lipofectamine^®^ 2000 Transfection Reagent (Invitrogen, San Francisco, CA, USA). Thirty-nanomolar miR-198 mimic or negative control, 100-nanomolar siRNA were used for transfection in Opti-MEM serum-free medium (Gibco, San Francisco, CA, USA), respectively. Total RNA and protein were prepared at 24 or 48 h after transfection and further used for qPCR or Western Blot analysis.

### 3.3. Quantitative Analysis of miRNAs and mRNAs

Total RNA was extracted from cultured cells using TRizol (Invitrogen, USA) according to the manufacturer’s protocols. The poly A tailed reverse transcription-polymerase chain reaction (RT-PCR) approach was used to assess the expression of miR-198 with kits from Ribo Bio (Guangzhou, China), and U6 expression was selected as internal reference. For quantitative analysis of CCND2 mRNA expression, 100–200 ng of total RNA was used for synthesis of random-primed single stranded cDNA using Primescript RT reagent kit (TaKaRa, Shiga, Japan) and cDNA was subjected to quantitative PCR using SYBR green Supermix (Bio-Rad, San Francisco, CA, USA) The relative amount of CCND2 mRNA transcripts was normalized to GAPDH. The sequence of oligonucleotides used as PCR primers were: GAPDH (forward) 5′-GGATGATGTTCTGGAAGAGCC-3′, GAPDH (reverse) 5′-AACAGCCTCAAGATCATCAGC-3′; CCND2 (forward) 5′-CGCAAGCATGCTCAGACCTT-3′, CCND2 (reverse) 5′-TGCGATCATCGACGGTGG-3′. Three independent experiments were performed in triplicates.

### 3.4. Protein Extraction and Western Blot

Twenty-four hours after transfection, HaCaT cells was lysed using the cell lysis buffer containing 1 mM phenylmethylsulfonylfluoride (Beyotime, Beijing, China). Protein concentration was determined with the BCA Protein Assay kit (Beyotime, China) and equal amounts of total protein were separated in 10% sodium dodecyl sulfate (SDS)-polyacrylamide gels, transferred to polyvinylidenedifluoride membranes (Bio-Rad, USA). Membranes were blocked for 1 h with 5% BSA in Tris-buffered saline containing 0.05% Tween 20, incubated overnight with primary antibody, washed and incubated with secondary antibody, and visualized by chemiluminescence (BeyoECLPlus, Beyotime, China). The antibodies used were as follows: mouse anti-GAPDH (AG019, Beyotime, China), mouse anti-CCND2 (BA2347-2, BOSTER, Wuhan, China), Horse Radish Peroxidase (HRP)-abeled Goat Anti-Mouse Immunoglobulin G (IgG) (H + L) (A0216, Beyotime, China).

### 3.5. Luciferase Assay

Prediction for the possible binding sites of miR-198 in the 3′-UTR of CCND2 mRNA was carried out using different online software, Pic Tar, Target Scan and the miR DB. Fractions of the 3′-UTR of CCND2 mRNA containing the wide-type or the mutated predicted binding sites were prepared and cloned downstream firefly luciferase in the pMIR-REPORT™ vector (Applied Biosystems, San Francisco, CA, USA). The mutation of the predicted binding sites was created using PCR Site Directed Mutagenesis. Cells plated on 24-well plates were individually transfected with 100 ng of pMIR-REPORT-CCND2, pMIR-REPORTRβ-gal, and pMIR-REPORT™ and 30 nM miR-198 mimic or its negative control. After 24 h, cells were lysed and assayed with Luciferase Reporter Gene Assay Kit as well as *in situ* β-galactosidase Staining Kit (Beyotime, China) according to the manufacturer’s instructions. Three independent experiments were performed in triplicate.

### 3.6. Cell Proliferation

For determination of cell viability by CCK-8 (Cell Counting Kit-8, Dojindo, Kumamoto-ken, Japan) assay, HaCaT cells were suspended in RIPM 1640 containing 10% fetal bovine serum and cultured in 96-well (5 × 10^3^/100 μL) plates. After transient transfection with miR-198 mimic, negative control oligonucleotides or CCND2 siRNA, respectively, cell proliferation ability was evaluated at 24 or 48 h using the CCK-8 assay. The colorimetric assay was read at 450 nm in a Microplate Reader (SpectraMax M2, Molecular Devices, Silicon Valley, CA, USA). All experiments were performed in triplicate and repeated twice. The cell cycle of transfected cells were also analyzed following double staining with AnnexinV-fluorescein isothiocyanate (FITC) and propidium iodide (PI) by flow cytometry (FCM).The data were plotted as means ± S.D. of three separate experiments.

### 3.7. Statistical Analysis

All experiments were repeated in triplicate and ANOVA was used for group comparisons. SPSS 10.0 (SPSS, Chicago, IL, USA) was used for data analysis. Statistical significance was defined as *p* value <0.05.

## 4. Conclusions

In conclusion, our results show that miR-198 represses HaCaT cell proliferation, and leads to significant cell cycle repression. As a new target gene of miR-198, the expression of CCND2 mRNA and protein are both downregulated by miR-198. In addition, the deficiency of CCND2 showed similar inhibition of HaCaT cell proliferation and cell cycle progression. All these data indicate that miR-198 inhibits HaCaT cell proliferation by targeting CCND2.
